# The Influence of Emotion Regulation on Estimation Strategy Execution in Individuals with Trait Anxiety

**DOI:** 10.3390/brainsci12091204

**Published:** 2022-09-07

**Authors:** Huan Song, Chenghui Tan, Chuanlin Zhu, Dianzhi Liu, Wenbo Peng

**Affiliations:** 1 School of Educational Science, Neijiang Normal University, Neijiang 641100, China; 2 Key Laboratory of Applied Psychology, Chongqing Normal University, Chongqing 401331, China; 3 Department of Psychology, Renmin University of China, Beijing 100872, China; 4 School of Educational Science, Yangzhou University, Yangzhou 225002, China; 5 School of Education, Soochow University, Suzhou 225002, China

**Keywords:** trait anxiety, negative emotion, emotion regulation, cognitive reappraisal, estimation strategy execution

## Abstract

Previous studies have shown that some negative emotions hinder estimation strategy execution. However, these studies rarely investigate the influence of negative emotions on the estimation strategy execution in individuals with trait anxiety. The present study examines the relationship between negative emotions and trait anxiety in individuals’ estimation strategy execution. Moreover, it looks into the influence of different emotion regulation strategies on their estimation strategy execution. In October 2010, 803 college students were evaluated using the Trait Anxiety Scale. From these participants, individuals with high and low trait anxiety were selected to complete the double-digit multiplication estimation task. The results showed that the estimation strategy’s execution speed in individuals with high trait anxiety was slower than those with low trait anxiety under negative emotions (*t* (113) = −2.269, *p* = 0.025, *d* = 0.427). Both expression inhibition and cognitive reappraisal could significantly improve the execution speed of the estimation strategy in low trait anxiety (*p* < 0.001). For individuals with high trait anxiety, cognitive reappraisal regulating negative emotions can promote the estimation strategy’s execution speed (*p* = 0.031). However, the use of expression inhibition has no significant effect on estimation strategy execution (*p* = 0.101). In summary, the present study revealed that different emotion regulation strategies moderated the arithmetic strategy execution of individuals with trait anxiety, and cognitive reappraisal had a better effect in individuals with high trait anxiety.

## 1. Introduction

Estimation, as an important part of constructing individual mathematical cognition, has been paid much attention by researchers in the previous literature. Estimation is the use of computing skills in a relatively short period of time [1]. This is performed without an accurate calculation to keep the estimation results more reasonable. Therefore, speed is an important measure of estimation. Estimation strategies refer to certain rules or procedures that people use in order to obtain estimation results faster and more accurately [2,3]. Considering the multiplication estimation task as an example, the commonly used estimation strategies include the round-up strategy (RU), the round-down strategy (RD), and the up-down strategy (UD) and the down-up strategy (DU) [4,5]. Strategy execution is a process in which an individual combines a given task and uses a given strategy to solve a problem [6]. It mainly includes the execution’s speed and accuracy [7]. Strategy execution is a good measure to examine how individuals utilize estimation strategies. Such a measure can likewise reflect the strategy’s very characteristics [8]. In previous studies, researchers have examined how emotion and emotion regulation strategies affect estimation strategy execution.

Studies have found that the influence of emotions on estimation strategy execution is mainly manifested as that estimation strategy execution promoted by positive emotions, such as happiness. On the other hand, the same is hindered by negative emotions, such as fear [9,10,11]. To reduce the impact of negative emotions on individual cognitive tasks, researchers began to explore whether emotion regulation under negative emotions could affect the individual estimation strategy execution. This is performed to reduce the influence of negative emotions on estimation strategy execution. Hu investigated from the perspective of habitual and non-habitual emotion regulation strategies. He found that under negative emotions (i.e., sadness), individuals using habitual emotion regulation strategies responded faster than non-habitual emotion regulation strategies to complete estimation tasks [12]. Another study showed that both cognitive reappraisal and expression suppression could improve individual arithmetic performance, and the regulation effect of cognitive reappraisal was greater than that of expression suppression [13]. Emotion regulation pertains to individuals attempting to adjust their inner experience, psychological state, and behavior performance by changing their emotional response’s intensity, direction, and duration to achieve the expected goal [14,15]. Expression suppression and cognitive reappraisal are two well-recognized, effective, and widely used emotion regulation strategies [16,17,18]. The former occurs after the emotional reaction tendency appears. Here, the individual actively hides and suppresses the corresponding emotions’ external performance to reduce their subjective emotional experience [11]. The latter is to adjust one’s emotional experience by changing the way individuals judge the situation subjectively, reconstructing the situation’s meaning and its emotional impact [19].

Previous studies on estimation strategy execution have their own emphasis. However, only a few studies have examined how emotion and emotion regulation strategy affect estimation strategy execution for individuals with trait anxiety. Adolescence is a risk period for anxiety, and long-term emotional disorders may lead to psychiatric disorders in adolescents [20,21]. The survey in China found that college students’ trait anxiety detection rate is 49.50%, which needs to be paid attention to [22]. Trait anxiety refers to individuals’ tendency to evaluate internal stimuli or external events in a way that causes anxiety [23]. In a study that compared individuals with trait anxiety, it was found that those with high trait anxiety performed worse in estimating strategy execution. This result was manifested as a slower response speed in estimating strategy execution [24]. This can be explained by processing the efficacy theory. Trait anxiety will occupy part of the limited working memory resources, and the resources used for the current cognitive tasks will be reduced [5,25], so that the individual execution speed of the estimation policy will be slowed down due to resource contention. However, for individuals with high trait anxiety, stable personality traits will affect their emotional feelings and emotional regulation. This makes them habitually maintain a high level of anxiety [26,27].

In summary, previous studies have found that some negative emotions can hinder estimation strategy execution [11], and cognitive reappraisal and expression suppression can reduce this obstacle [12,13], but few researchers have focused on college students with trait anxiety. Therefore, this study consists of two experiments. First, college students were tested to investigate the difference negative emotions’ influence has on estimation strategy execution for individuals with high and low trait anxiety (experiment 1). Second, the influence of different emotion regulation strategies on estimation strategy execution in individuals with trait anxiety was further examined (experiment 2). The two experiments are expected to provide guidance for improving the estimation strategy execution for college students with trait anxiety affected by negative emotions.

## 2. Experiment 1: The Influence of Negative Emotions on the Estimation Strategy Execution in Individuals with High and Low Trait Anxiety

### 2.1. Participants

In October 2021, a cluster sample of 803 college students, including 251 males and 552 females, was conducted in a college in Neijiang by using Trait Anxiety Scale. Referred to previous studies [28], the way to determine high and low anxiety participants was based on the pre-and post-percentiles of a certain anxiety score, and the percentiles of trait anxiety scale scores were calculated. The top 27% of the scores (≥48) were the high trait anxiety group, and the bottom 27% of the scores (≤39) were the low trait anxiety group. An independent sample *t*-test investigation showed that there was a significant difference between the high trait anxiety group (52.87 ± 3.73) and the low trait anxiety group (34.72 ± 4.82), *t* (481) = −46.351, *p* < 0.001, *d* = 4.227. Following the voluntary principle, the participants with high and low trait anxiety were invited to participate in the experiment. A total of 115 participants were recruited (low trait anxiety group: 22 males and 35 females; high trait anxiety group: 25 males and 33 females), aged 20.21 ± 1.12 (M ± SD) years. The independent sample *t*-test results showed that there were significant differences between the high trait anxiety group (52.64 ± 4.75) and the low trait anxiety group (34.19 ± 3.47), *t* (113) = −23.744, *p* < 0.001, *d* = 4.467. All participants were right-handed and had normal or corrected-to-normal vision. They signed an informed consent form before we began the experiment and were paid 8 CNY after the experiment. This study was approved by the Ethics Committee of our school and conformed to ethical standards.

### 2.2. Design

A univariate between-subjects factorial design was employed in experiment 1. The group (high trait anxiety group vs. low trait anxiety group) was used as the independent variable, and the emotional experience intensity, the accuracy, and reaction times (RTs) of estimation strategy execution were used as the dependent variables.

### 2.3. Materials

#### 2.3.1. Trait Anxiety Scale

The trait anxiety subscale of State–Trait Anxiety Scale developed by Spielberger et al. is used to evaluate a relatively stable anxiety tendency with individual differences [29]. The Chinese revised version of the scale was adopted in this study [30] to assess the level of trait anxiety of participants in the current study. The subscale has 20 items and is answered on a 4-point Likert scale ranging from “absolutely not” to “very evident”; a high score indicates a high degree of trait anxiety. The internal consistency coefficient of the scale in this study was 0.877.

#### 2.3.2. Positive and Negative Affect Scale (PANAS)

PANAS [31] includes two subscales, that is, PA and NA, where PA measures the participants’ positive emotions and NA measures their negative emotions. There are 10 items each for PA and NA, each of which is scored on a 5-point Likert scale. The internal consistency coefficients of PA and NA in this study were 0.76 and 0.93, respectively.

#### 2.3.3. Emotional Pictures

A total of 50 pictures with clear content and clear meaning were selected from the Chinese Affective Picture System (CAPS) [32], were used to stimulate the subjects’ emotions, such as sadness, disgust, fear, and anger, of which 5 were used as practice materials and 45 were used as formal experiment materials. The average value of the pictures was 2.57 (SD = 0.54) and the average arousal was 5.74 (SD = 0.71).

#### 2.3.4. Multiplication Estimation Problems

The rounding-up strategy (RU), rounding-down strategy (RD), up-down strategy (UD), and down-up strategy (DU) are the four most common estimation strategies [4,5]. Take the multiplication estimation task as an example: the RU strategy is to up-regulate both operands to the nearest decade; the RD strategy is to down-regulate both operands to the nearest decade; the UD strategy is to up-regulate the multiplicand and down-regulate the multiplier, respectively, to the nearest decade (e.g., 23 × 47 to estimate 30 × 40); the DU strategy is to down-regulate the multiplicand and up-regulate the multiplier, respectively, to the nearest decade [33,34]. Studies have found that RD strategy is the simplest, RU and UD strategy are both difficult, because RU strategy needs to increment the two operands and keep them in working memory, while DU and UD strategies need to switch the operands [27,35]. Previous studies have found that Chinese participants have a multiplier order effect [36], that is, compared with the formula that the second multiplier is smaller than the first multiplier, when the second multiplier is larger than the first multiplier, the participants’ scores are worse. For example, the performance of calculating 37 × 35 is worse than that of calculating 35 × 37. Therefore, this study selected the DU strategy that is more difficult and accords with the priority of smaller operands, in other words, all estimation problems in this study follow that the second multiplier is larger than the first multiplier, for example, 27 × 64, excluding 64 × 27.

According to previous studies [11,37], the selection of multiplication estimation problems follows the following principles: (1) all the tens of the operand are not digit 0 or 5, for example, 21 × 30, 25 × 37, 20 × 40, 25 × 45, and so on are not included in the problems; (2) no operand included the tens digit 1 or 9 (e.g., 13 × 38, 42 × 98, 12 × 98, etc.); (3) the tens digit of all multipliers is not the same (e.g., 51 × 57); (4) no operands included a repeated digit, for example, it does not contain the equations 22 × 47, 31 × 55; (5) no problems included the same operand (e.g., 34 × 34, 46 × 46); (6) exclude the calculation formulas with the same operand after rounding using the DU strategy in two adjacent trials, such as 36 × 72 and 37 × 74.

### 2.4. Procedure

**Figure 1:**
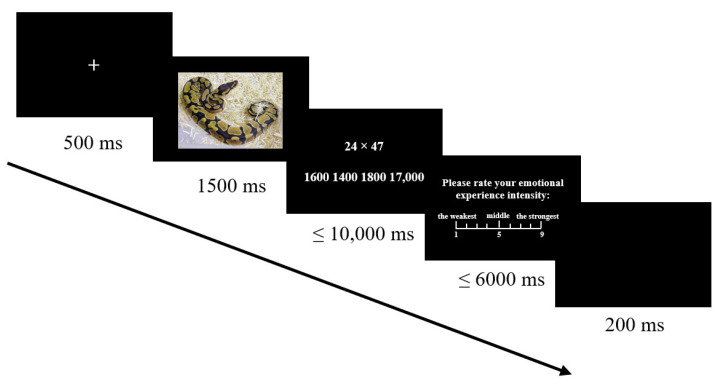
Flow chart of experiment 1.

The experiment program was generated and presented using E-prime 2.0 (Psychology Software Tools, Inc., Sharpsburg, PA, USA). To exclude the possible interference caused by the participants’ emotions in their natural state [38,39], PANAS was used to score the participants’ emotions prior to the experiment. Before the formal experiment, the participants practiced for 5 trials, during which feedback was provided, so that they could clearly understand the experimental process. The negative emotional pictures and estimation problems in the practice stage would not repeatedly present in the formal experiment. A total of 45 estimation problems suitable for using the DU strategy were selected in this experiment. The font for the estimation task was 58, and the font for alternative answer, such as emotional rating, was 24. A total of 1 block of 45 trials were presented in white font with a standard black background. In a quiet laboratory, participants were instructed to adjust the chair, about 40 cm away from the screen. First, the instructions were presented, participants could move on to the practice stage by pressing the “Enter” key if they understood the instructions well. Then, participants were asked if they wanted to enter the formal experiment after finishing practice. As shown in Figure 1, a “+” fixation lasting 500 ms is presented at first in the formal experiment, then randomly presented a negative picture for 1500 ms. Participants were asked to watch the emotional picture and feel their emotions. Next, the multiplicative estimation task was presented at the top center of the screen, with four alternative answers presented side by side at the bottom of the screen. Participants used “D”, “F”, “J”, and “K” key to select the correct answer from left to right according to the DU strategy. The correct answers are balanced so that they are equally likely to appear in all four places. In the multiplicative estimation task, the participants were required to complete the estimation task quickly and accurately within 10,000 ms. Then, they were asked to rate their immediate emotional feelings using a 9-point scale (1 = no feeling at all, 5 = medium, 9 = very strong feeling) [40]. If the button was not pressed for more than 6 s, the trial was invalid and entered the next trial. All statistical analyses were conducted using SPSS 22.0 (SPSS Inc., Chicago, IL, USA).

### 2.5. Results

#### 2.5.1. Additional Variable and the Assessment of Emotional Experience Intensity

The independent sample *t*-test was used to analyze the results of the two groups of subjects on the two subscales of the PANAS, namely the PA and NA. In the PA score, there was no significant difference between the low trait anxiety group (31.96 ± 4.14) and the high trait anxiety group ((31.14 ± 4.83), *t* (113) = 0.986, *p* = 0.326, *d* = 0.186). Furthermore, there was no significant difference in the NA score between the low trait anxiety group (20.75 ± 3.21) and the high trait anxiety group ((21.67 ± 4.38), *t* (113) = −1.281, *p* = 0.203, *d* = 0.241).

The independent sample *t*-test results showed that there was no significant difference between the subjective emotional scores in the high (5.26 ± 1.17, M ± SD) and low trait anxiety (5.31 ± 1.17, M ± SD) groups, *t* (113) = −0.230, *p* = 0.819, *d* = 0.043. This indicated that individuals with high and low trait anxiety experience similar subjective emotions when watching negative emotional pictures.

#### 2.5.2. The Accuracy and RTs of Estimation Strategy Execution

In addition, an independent sample *t*-test was performed to test the group difference between the individuals with high and low trait anxiety in the effect of negative emotion on the accuracy and RTs of estimation strategy execution. The results found that under negative emotions, there was no significant difference between the low trait anxiety group (94.55 ± 3.29, M ± SD) and the high trait anxiety group (94.40 ± 3.65, M ± SD) on the accuracy of the estimation strategy execution, *t* (113) = 0.232, *p* = 0.817, *d* = 0.044. However, there was a significant group difference in the RTs of the estimation strategy execution between the individuals with low (3322.25 ± 523.09, M ± SD) and high trait anxiety (3588.64 ± 718.69, M ± SD) under negative emotion, *t* (113) = −2.269, *p* = 0.025, *d* = 0.427. That is, the response speed of the estimation strategy execution in the low trait anxiety group was significantly faster than that in the high trait anxiety group.

### 2.6. Discussion

Experiment 1 examined the group difference in the estimation strategy execution between the individuals with high and low trait anxiety affected by negative emotions. The results showed that there was no significant group difference in the estimation strategy execution’s accuracy under negative emotion. This may be explained by the fact that the ceiling effect occurs in the present study because the accuracy measure is not sensitive to the study of estimation strategy with college students as participants. The same result has also been found in previous studies [5,11,41]. However, with negative emotions, the estimation strategy’s execution speed in individuals with high trait anxiety was slower than those with low trait anxiety. On the one hand, the processing efficiency theory holds that anxiety itself will occupy part of the limited working memory resources. The same will in turn reduce the resources used for the current cognitive task, leading to the decline in an individual’s processing efficiency due to resource competition [5,25]. Studies have found that individuals with high trait anxiety allocated more attention resources to negative emotional stimuli [42]. Therefore, negative emotions have a greater impact on estimation strategy execution in individuals with high trait anxiety. On the other hand, the attentional control theory suggested that anxiety will first hinder the individual’s processing efficiency (RTs), rather than the cognitive tasks performance (accuracy). This study was a double-digit multiplication estimation task requiring less attention and control resources. More attention and control resources were needed to complete the target task for individuals with high trait anxiety. Therefore, there was no difference in the estimation task performance between individuals with high and low trait anxiety. However, the processing efficiency of completing tasks will be reduced [43].

Studies have found that both cognitive reappraisal and expression suppression can help improve individual arithmetic performance, and the regulation effect of cognitive reappraisal was greater than that of expression suppression [13]. This is true when compared with non-habitual emotion regulation strategies. Thus, on the basis of experiment 1, experiment 2 will investigate whether the use of different emotion regulation strategies in regulating negative emotions can promote the estimation strategy execution for individuals with high and low trait anxiety. It will likewise look into whether there are group differences in the promotion effects.

## 3. Experiment 2: The Influence of Emotion Regulation Strategies on the Estimation Strategy Execution in Individuals with High and Low Trait Anxiety

### 3.1. Participants

Among the college students with high and low trait anxiety selected in experiment 1, those who had already participated in experiment 1 were excluded. Finally, 59 valid participants (low trait anxiety group: 10 males and 19 females; high trait anxiety group: 14 males and 16 females) were invited to participate in experiment 2 according to the principle of voluntariness, aged 20.29 ± 1.15 (M ± SD) years. The independent sample *t*-test results showed that there were significant differences between the high trait anxiety group (51.27 ± 2.61) and the low trait anxiety group (35.03 ± 2.78), *t* (57) = −23.105, *p* < 0.001, *d* = 6.122. All participants were right-handed and had normal or corrected-to-normal vision. They signed an informed consent form before we began the experiment and were paid 8 CNY after the experiment. This study was approved by the Ethics Committee of our school and conformed to ethical standards.

### 3.2. Design

A 2 (group: high trait anxiety group vs. low trait anxiety group) × 3 (emotion regulation strategy: free viewing vs. expression suppression vs. cognitive reappraisal) mixed factorial design was employed in experiment 2. The group was between-subjects factor, and the emotion regulation strategy was within-subjects factor. The emotional experience intensity, the accuracy, and RTs of estimation strategy execution were dependent variables.

### 3.3. Materials

#### 3.3.1. Trait Anxiety Scale, PANAS, Emotional Pictures, Multiplication Estimation Problems

Same as experiment 1.

#### 3.3.2. Emotion Regulation Strategy Questionnaire

By referring to previous methods of checking whether participants use emotion regulation strategies [44], the participants were asked to use 0–5 (0 = completely inconsistent, 5 = completely consistent) to score the real situation of their use of specified emotion regulation strategies. Under the expression suppression condition, the participants were asked: “During the experiment, did you try to keep your facial muscles as still as possible and try to hide your expression so as not to let others see your emotional feelings?” In the condition of cognitive reappraisal, participants were asked: “In the previous experiment, did you think positively, rationalizing the picture in a positive way, or imagining that the picture was irrelevant to you?”

### 3.4. Procedure

The procedure of experiment 2 was basically the same as experiment 1; the difference was that the instructions of emotion regulation strategy (free viewing, expression inhibition, cognitive reappraisal) were presented for 1000 ms before presenting the emotion picture. Participants were asked to follow the instructions to regulate negative emotion when watching the emotion picture. Studies have shown that instructions can be used to guide subjects to adopt different emotion regulation strategies [18,45]. Free viewing: please pay attention to observe the given picture and naturally feel your own emotions. Expression inhibition: please pay attention to the given picture, keep your facial muscles as still as possible in the process of feeling emotions, hide your expression as much as possible, do not show or let others see your emotional feelings. Cognitive reappraisal: please pay attention to the given picture and think differently or in a positive way, rationalize the picture in a positive way, such as “this is not true, this is a scene from a movie”. There were 15 practice trials and a total of 3 blocks of 45 trials in the formal experiment.

**Figure 2:**
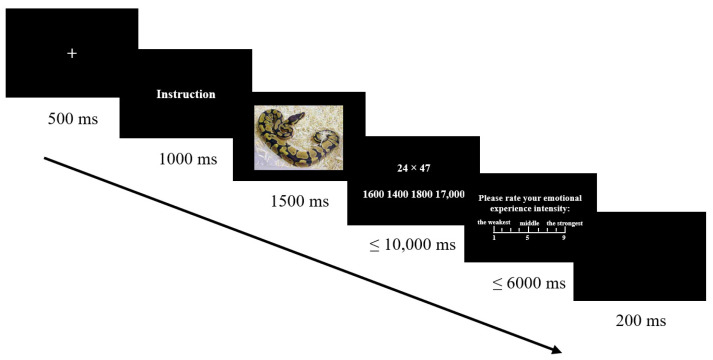
Flow chart of experiment 2.

In order to balance the order effect, two sets of procedures were used according to the type of emotion regulation strategy (free viewing, expression inhibition, and cognitive reappraisal). The first was free viewing–expression inhibition–cognitive reappraisal. The second was free viewing–cognitive reappraisal–expression inhibition. A total of 29 participants used the first set of procedures for the experiment, and the others used the second one. Before the end of each block, the instructions of emotion regulation strategy were presented again. The participants were required to rate the degree of compliance with the instructions in the experiment using a 4-point Likert scale (0 = completely inconsistent, 5 = completely consistent). There was a two-minute break between each block, see Figure 2 for the specific experiment procedure. All statistical analyses were conducted using SPSS 22.0 (SPSS Inc., Chicago, IL, USA). For the degree of freedom that did not satisfy the spherical test hypothesis, the Greenhouse–Geisser correction was applied to the p values, and all post hoc tests included Bonferroni’s correction.

### 3.5. Results

#### 3.5.1. Additional Variable and Manipulation Check

The independent sample *t*-test was used to analyze the results of the two groups of subjects on the two subscales of the PANAS, that is, the PA and NA. In terms of the PA score, there was no significant difference between the low trait anxiety group (30.10 ± 3.32) and the high trait anxiety group ((29.63 ± 3.99), *t* (57) = 0.491, *p* = 0.626, *d* = 0.130). In addition, there was no significant difference in the NA score between the low trait anxiety group (19.79 ± 3.91) and the high trait anxiety group ((20.77 ± 4.10), *t* (57) = −0.933, *p* = 0.355, *d* = 0.246).

In the experiment, the participants were asked to rate how well they regulated their emotions according to the given emotion regulation strategy. The results of the one-sample *t*-test showed that both the implementation of expression inhibition (4.44 ± 0.70; *t* (58) = 15.775, *p* < 0.001, *d* = 4.144) and cognitive reappraisal (4.27 ± 0.74; *t* (58) = 13.216, *p* < 0.001, *d* = 3.469) were significantly higher than the median of 3, respectively. Furthermore, the results of the 2 (group) × 3 (emotion regulation strategy) repeated-measures ANOVA showed that the main effect of the group (F (1,114) = 3.162, *p* = 0.081, $\eta^{2}$ = 0.053) and emotion regulation strategy (F (2,114) = 3.189, *p* = 0.079, $\eta^{2}$ = 0.053) were not significant, nor was the interaction effect (F (2,114) = 0.001, *p* = 0.976, $\eta^{2}$ = 0.000). This indicated that the participants with high and low trait anxiety effectively used the corresponding strategies to regulate their negative emotions as instructed, and there was no group difference in the effectiveness of the different strategies used.

#### 3.5.2. The Assessment of Emotional Experience Intensity

The results of the 2 (group) × 3 (emotion regulation strategy) repeated-measures ANOVA showed that the main effect of the emotion regulation strategy was significant (F (2,114) = 35.992, *p* < 0.001, $\eta^{2}$ = 0.387), and the post hoc analysis showed that the emotional experience intensity after using expression inhibition and cognitive reappraisal were lower than free viewing (*p* < 0.001), while there was no significant difference between using expression inhibition and cognitive reappraisal (*p* > 0.05). In addition, the main effect of the group was significant (F (1,57) = 5.996, *p* = 0.017, *2* = 0.095); the emotional experience intensity in the high trait anxiety group (4.44 ± 0.20) was lower than low trait anxiety group (3.72 ± 0.21). The interaction effect was significant, F (2,114) = 3.240, *p* = 0.043, $\eta^{2}$ = 0.054, and the simple-effect analysis showed that the emotional experience intensity after using expression inhibition (3.04 ± 1.70) and cognitive reappraisal (3.13 ± 1.61) were lower than free viewing (5.00 ± 0.84) in the low trait anxiety group (*p* < 0.001), while there was no significant difference between using expression inhibition and cognitive reappraisal (*p* > 0.05). The results were similar for the high trait anxiety group, and the emotional experience intensity after using expression inhibition (4.25 ± 1.74, *p* < 0.01) and cognitive reappraisal (3.89 ± 1.78, *p* < 0.001) were lower than free viewing (5.17 ± 0.36), while there was no significant difference between using expression inhibition and cognitive reappraisal (*p* > 0.05).

#### 3.5.3. The Accuracy and RTs of Estimation Strategy Execution

**Figure 3:**
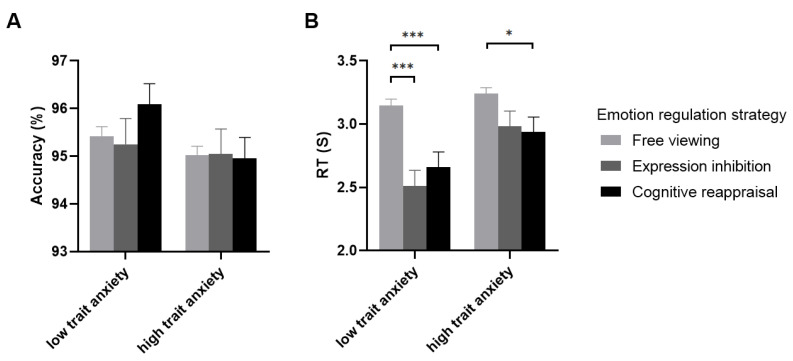
The accuracy (**A**) and RTs (**B**) of estimation strategy execution in the low and high trait anxiety groups after using free viewing, expression inhibition, and cognitive reappraisal to regulate negative emotions.“*” means “*p* < 0.05”, “***” means “*p* < 0.001”.

We analyzed the accuracy and RTs of the estimation strategy execution with the 2 (group) × 3 (emotion regulation strategy) repeated-measures ANOVA (see Figure 3). The results of the accuracy showed that the main effect of the group (F (1,57) = 2.540, *p* = 0.117, $\eta^{2}$ = 0.043) and emotion regulation strategy (F (2,98) = 0.523, *p* = 0.594, $\eta^{2}$= 0.009) were not significant, nor was the interaction effect (F (2,98) = 0.746, *p* = 0.477, $\eta^{2}$ = 0.013). However, the results of the RTs indicated that the main effect of the emotion regulation strategy was significant (F (2,84) = 22.370, *p* < 0.001, $\eta^{2}$ = 0.282), and the post hoc analysis showed that the RTs of the estimation strategy execution after using expression inhibition (2746.67 ± 86.58, *p* < 0.001) and cognitive reappraisal (2797.41 ± 84.97, *p* < 0.001) were faster than free viewing (3192.99 ± 35.16), while there was no significant difference between using expression inhibition and cognitive reappraisal (*p* > 0.05). The main effect of the group was also significant (F (1, 57) = 5.557, *p* = 0.022, $\eta^{2}$ = 0.089), and the RTs in the high trait anxiety group (3052.57 ± 83.40) were slower than in the low trait anxiety group (2772.15 ± 84.83). The interaction effect was significant, F (2,84) = 3.346, *p* = 0.039, $\eta^{2}$ = 0.055, and the simple-effect analysis showed that the RTs after using expression inhibition (2511.56 ± 123.48) and cognitive reappraisal (2657.98 ± 121.18) were faster than free viewing (3146.90 ± 50.14) in the low trait anxiety group (*p* < 0.001), while there was no significant difference between using expression inhibition and cognitive reappraisal (*p* > 0.05). For the high trait anxiety group, the RTs after using cognitive reappraisal (2936.84 ± 119.14) was faster than free viewing (3239.10 ± 49.30, *p* = 0.031), while there was no significant difference between using expression inhibition (2981.78 ± 121.40) and cognitive reappraisal (*p* > 0.05), and no significant difference between expression inhibition and free viewing (*p* > 0.05).

### 3.6. Discussion

Experiment 2 examined the effect of different emotion regulation strategies on the estimation strategy execution in individuals with high and low trait anxiety. The estimation execution strategy’s results suggested that individuals with high and low trait anxiety could not improve the estimation execution strategy’s accuracy by using cognitive reappraisal and expression inhibition to regulate negative emotions. Specifically, both cognitive reappraisal and expression inhibition could promote the estimation strategy’s execution speed for individuals with low trait anxiety. On the other hand, cognitive reappraisal was better than expression inhibition in promoting the estimation strategy’s execution speed for individuals with high trait anxiety.

As mentioned earlier, the cognitive reappraisal strategy is used to regulate negative emotion to promote the speed of the estimation strategy execution in individuals with trait anxiety. This is used because cognitive reappraisal is an advanced strategy that occurs before the emotional response. Specifically, it changes the individual’s subjective judgment of negative emotions to regulate their emotions. Conversely, expression inhibition mainly reduces the individual’s subjective emotional experience. It actively hides and suppresses the external performance of emotions, which occurs after the emotional response [46]. In other words, cognitive reappraisal can reduce an individual’s negative emotional experience at an early stage and does not require long-term efforts. In contrast, expression inhibition requires positive efforts to suppress emotional responses [47]. Expression inhibition will cost more cognitive resources. Individuals with high trait anxiety will have less cognitive resources for the current estimation task. Thus, this will slow down the estimation strategy’s execution speed. Therefore, using cognitive reappraisal in regulating negative emotions, rather than expression inhibition, can more effectively promote the estimation strategy execution’s speed for individuals with high trait anxiety.

## 4. General Discussion

In this study, we used negative images depicting daily life situations as materials. We then combined the same with the emotional priming paradigm to investigate the differences in the influence of negative emotions and emotion regulation strategies on estimation strategy execution in individuals with high and low trait anxiety. Combining our findings with the results of experiment 1 and experiment 2, it can be shown that individuals with high and low trait anxiety experience similar negative emotions when observing the emotional pictures. Both cognitive reappraisal and expression inhibition could effectively regulate their negative emotions. Moreover, there was no group difference in the regulation effect. This suggested that individuals with high and low trait anxiety have similar emotional experience intensity and regulation effects in using different emotional regulation strategies. Therefore, this finding is consistent with a previous study [48]. However, its effect on cognitive activity may vary depending on the trait anxiety’s level. The estimation task accuracy’s results indicated that negative emotion and different emotion regulation strategies had no influence on the estimation strategy execution’s accuracy in individuals with high and low trait anxiety. As mentioned above, the reason may be that the estimation strategy is less difficult for college students. This leads to the insensitivity of the estimation strategy execution’s accuracy index. Therefore, it is necessary to consider the estimation strategy problems suitable for different samples in the future.

It is worth noting that through two experiments, we found that negative emotions and emotion regulation strategies had different effects on the estimation strategy’s execution speed for individuals with high and low trait anxiety. Specifically, the estimation strategy’s execution speed in individuals with low trait anxiety was significantly faster than those with high trait anxiety under negative emotions. Individuals with high trait anxiety, when faced with negative emotional stimuli, allocated more attention resources to irrelevant stimuli due to a reduced inhibitory control ability. This in turn led to the decrease in attention resources for the ongoing cognitive task. Therefore, it affected the estimation strategy’s execution speed. After using emotion regulation strategies in regulating negative emotions of individuals with trait anxiety, it will reduce the attention resources allocated to the irrelevant stimuli. Thus, it promotes their estimation strategy’s execution speed. Based on this finding, experiment 2’s results showed that both cognitive reappraisal and expression inhibition used to regulate negative emotion could promote the estimation strategy’s execution speed in individuals with low trait anxiety. On the other hand, the use of cognitive reappraisal had a better promoting effect for individuals with high trait anxiety. In other words, although expression inhibition can effectively regulate the negative emotions of individuals with high trait anxiety, it is difficult to promote their estimation strategy’s execution speed after using expression inhibition to regulate negative emotions. The possible reason is that individuals with high trait anxiety need to make more efforts to suppress their emotional response. Moreover, the use of expression inhibition will occupy more cognitive resources. Thus, it is difficult to promote the estimation strategy’s execution speed due to a lack of cognitive resources used on the current task. Some studies have found that college students with high trait anxiety tend to use expression inhibition [49] rather than cognitive reappraisal [48]. This suggested that the use of cognitive reappraisal to regulate negative emotions for individuals with high trait anxiety may have a better effect on their cognitive activities.

The results need to be interpreted with caution given that several limitations were present in this study. One limitation was that the DU strategy with greater difficulty was selected in this study. However, the same was easier for college students. In future studies, primary and middle school students could be considered as participants to increase the estimation strategies’ difficulty. Moreover, it will allow researchers to further investigate the influence of emotion and emotion regulation on estimation strategy execution. Another limitation is that there was only a single type of estimation strategy in our study. Particularly, it only included the DU strategy selected. Whether our findings could be extended to other types of estimation strategies remains to be further studied. Studies have shown that strategy difficulty affects estimation strategy execution [50]. Furthermore, future research could take the estimation strategy’s difficulty into consideration. Finally, the present study adopted the negative pictures depicting common scenes in daily life. While it contained a variety of negative emotions and was more ecological, it did not distinguish between different types of negative emotions. Thus, there is a need for more systematic studies to investigate the influence of the different types of negative emotions and emotion regulation strategies on estimation strategy execution in individuals with trait anxiety.

## 5. Conclusions

In conclusion, this study revealed that the estimation strategy’s execution speed in individuals with low trait anxiety was significantly faster than those with high trait anxiety under negative emotions. Furthermore, both cognitive reappraisal and expression inhibition used to regulate negative emotions could promote the estimation strategy’s execution speed in individuals with low trait anxiety. On the other hand, cognitive reappraisal had a better promoting effect for individuals with high trait anxiety.

This study investigated the influence of negative emotions on the estimation strategy execution of college students with high and low trait anxiety and the manner in which the negative emotions of college students with trait anxiety can be effectively regulated to promote estimation strategy execution and help individuals with trait anxiety to choose a greater emotion regulation strategy. Simultaneously, it provides a reference for teachers to improve the implementation of the estimation strategy of trait anxiety individuals, which has certain enlightenment significance for the students and educators.

## Data Availability

Not applicable.
